# From Mood Swings to Psychosis: Exploring the Psychiatric Side Effects of Corticosteroids

**DOI:** 10.1192/j.eurpsy.2025.1855

**Published:** 2025-08-26

**Authors:** A. Pedro Costa, M. Mousinho, A. Matos Pires, G. Simões, A. I. Gomes, A. R. Coelho

**Affiliations:** 1 Departamento de Saúde Mental, Unidade de Saúde do Baixo Alentejo, Beja; 2 Departamento de Psiquiatria e Saúde Mental, Unidade Local de Saúde da Região de Aveiro, Aveiro; 3 Serviço de Oncologia Médica, Instituto Português de Oncologia de Coimbra Francisco Gentil, Coimbra, Portugal

## Abstract

**Introduction:**

Corticosteroids are integral in treating various medical conditions across multiple specialties. However, they are known to induce psychiatric adverse effects, ranging from subtle mood changes and memory deficits to psychosis.

**Objectives:**

This review aims to explore the current literature on these effects, identifying risk factors and strategies for early intervention.

**Methods:**

A non-systematized literature review was carried out on PubMed and Google Scholar. The following words were searched: (“corticosteroids” OR “steroids” OR “glucocorticoids”) AND (“psychiatry symptoms” OR “psychosis” OR “mood” OR “memory”).

**Results:**

Despite being recognized for their strong anti-inflammatory and immune-suppressing effects across various medical conditions, corticosteroid therapies frequently come with neuropsychiatric complications, the understanding of which is still limited. Although symptoms usually emerge within 3 to 4 days after starting corticosteroid treatment, they can manifest at any point, even after the therapy has been completed or stopped. Dosage is a significant risk factor, with high doses increasing the likelihood of psychosis. Depression is more prevalent among women. Additional risk factors include past psychiatric history, compromised blood-brain barrier, and hypoalbuminemia. However, some instances show beneficial outcomes, such as alleviation of depressive symptoms without triggering mania and improvements in cognitive function.

**Conclusions:**

Early diagnosis and awareness are crucial in managing corticosteroid-induced psychiatric symptoms. Initial steps should involve tapering or discontinuing corticosteroids, supplemented by psychotropic medications if necessary.

**Disclosure of Interest:**

None Declared

